# Evolution of blood pressure from adolescents to youth in salt sensitivies: a 18-year follow-up study in Hanzhong children cohort

**DOI:** 10.1186/1475-2891-11-70

**Published:** 2012-09-14

**Authors:** Jianjun Mu, Shuhui Zheng, Qiufang Lian, Fuqiang Liu, Zhiquan Liu

**Affiliations:** 1 Department of Cardiology, First Affiliated Hospital of Medical College, Xian Jiaotong University, Xian Shaanxi, 710061, P. R. China; 2 Dept. of Cardiovascular Medicine, First Affiliated Hospital, Xian Jiaotong University, Xian Shaanxi, 710061, P. R. China

**Keywords:** Blood pressure, Salt sensitivity, Risk factor, Adolescent

## Abstract

**Background:**

Essential hypertension mostly originates from children. Salt Sensitivity (SS) is regarded as the intermediate phenotype of essential hypertension. The present study investigated the effects of salt-sensitivity on evolution of blood pressure (BP) and development to hypertension from adolescents to youth.

**Methods:**

A baseline survey was carried out in 4,623 adolescents aged 6-15 years old in Hanzhong rural areas in 1987, 310 of whom(mean 9.2 years) were randomly recruited for determination of salt sensitivity using the tests of oral saline load and furosemide sodium-volume depletion. SS was diagnosed in 101 subjects while 209 were determined as non-salt-sensitive (NSS). We made a 18-year followed-up of the cohort in 2005.

**Results:**

The response rate for surviving baseline adolescents was 71.9%. At follow up, BP in youth with baseline SS was higher than that in NSS (SBP:122.9 ± 13.1 *VS* 117.3 ± 12.4, *P < 0.01;* DBP: 78.2 ± 10.4 *VS* 74.7 ± 10.8, P < 0.05). Longitudinal analysis of 18-year BP evolution, subjects in SS had greater Systolic BP change than subjects in NSS(19.6 ± 12.714.7 ± 12.2, *P* < 0.01). The incidence of hypertension in salt sensitive group was higher than that in NSS group (15.5% *VS* 6.3%, RR = 2.34, *P < 0.05).*

**Conclusion:**

Our findings indicate that adolescents with higher BP salt-sensitivity have a higher rate of incident hypertension in youth. Salt sensitivity could be at high risk predisposing to development of hypertension from adolescents to youth.

## Introduction

The prevalence of hypertension in China has been rising rapidly from 5.11% to 18.8% during the last 30 years [1].To study evolution of adolescents’ blood pressure and identify risk factors for hypertension is significantly important for effective prevention of essential hypertension, as well as alleviation of cardiovascular diseases [2,3].

Essential hypertension is a complex trait, influenced by multiple genetic and environmental factors. The epidemiological studies during the past century generally confirmed salt as an crucial environmental factor for development of hypertension [4]. A heterogeneous BP response to changes in dietary sodium chloride intake, a phenomenon generally referred to as salt-sensitivity, is observed in both hypertensive patients and normotensive individuals [5]. SS is currently regarded as the intermediate phenotype of essential hypertension. SS of BP has been associated with an increased risk of hypertension, cardiovascular disease and premature death [6,7]. There has been a 15-year follow-up study in adults indicated that subjects with higher BP salt-sensitivity have a higher rate of incident hypertension [8]. However, no studies reported for long-term follow up of blood pressure in adolescents with SS. This study aims to explore the effects of salt sensitivity on evolution of blood pressure and development to hypertension from adolescents to youth, by following up the subjects in the “Hanzhong cohort of adolescent hypertension study” recruited in 1987.

## Methods

### Cohort of study

In March and April, 1987, we established the cohort of Hanzhong Adolescent Hypertension Study [9,10]. Based on a baseline survey of 4,623 adolescents aged 6-15 years old in over 20 schools of three towns (Qili, Laojun and Shayan) in Hanzhong, Shaanxi, China. General information, medical history, blood pressure, height, body mass index, waistline, arm circumference, pulse, etc. were collected. BMI was calculated as kilograms per meters squared (kg/m2). Salt sensitivity was measured in a randomly selected sample of 310 adolescents using the tests of oral saline load and furosemide sodium-volume depletion [11]. Salt sensitivity (SS) was diagnosed in 101 subjects(mean 9.3 years)while 209 (mean 9.2 years)were determined as non-salt sensitive (NSS). The profile of baseline was exhibited in Table 1. There was no difference between the two groups in terms of age, family history of hypertension, body mass index, blood pressure, resting heart rate, etc.

**Table 1:** General information of SS and NSS adolescents at baseline in 1987 (x±s)

**Group**	**Sample No. (Male/Female)**	**Age (Year)**	**FH+ (%)**	**BMI (kg/m**^**2**^**)**	**SBP (mmHg)**	**DBP (mmHg)**	**HR (Beat/min)**
SS	101 (57/44)	9.3±1.9	20.8	15.9±1.3	102.8±10.2	65.8±9.8	80.7±8.8
NSS	209 (118/91)	9.2±1.9	18.7	15.5±1.2	101.9±11.4	65.1±9.5	79.4±9.5

SS, Salt Sensitive; NSS, Non-salt-sensitive; FH+, hypertension family history (positive); BMI, body mass index; SBP, systolic blood pressure; DBP, diastolic blood pressure; HR, heart rate.

We made a 18-years follow-up of the subjects in the above cohort in March and April, 2005. Through baseline survey and follow-up examinations, the subjects excluded the secondary hypertension patients and patients with other severe diseases. The baseline survey plan was approved by the Academic Committee of the First Affiliated Hospital of Xian Medical University, and parents of the surveyed adolescents had all signed the informed consent form. The follow up plan was approved by the Ethics Committee of the First Affiliated Hospital of Medical College, Xian Jiaotong University, and the followed subjects had all signed the informed consent form.

### Tests to determine SS

Oral saline load test and natriuretic test were used to determine salt sensitivity of the subjects. Fasting adolescents were sent to Shaanxi Hanzhong Cardiovascular Disease Institute in early morning. After 30 minutes of resting, their BP was measured for three times, the average of the three measurements taken as the “presaline” mean arterial blood pressure (MABP, it was derived from the following formula: diastolic pressure + 1/3 pulse pressure). The subjects then drank up a certain volume (in dose of 100 ml per age) of 1% saline within 30 minutes, and BP was again measured 2 hours after salt loading to be considered as “postsaline” MABP. Then natriuretic test were accomplished by administration of a 40 mg doses of furosemide just after BP measurement. The BP was for the third time measured 2 hours after and the “postfurosemide” MABP was calculated. If the summation from the increase of “postsaline” MABP 2 hours after salt loading comparing with preloading and the “postfurosemide” MABP decrease 2 hours after sodium depletion comparing with prefurosemide was equal to or exceeding 10 mm Hg, the subject was deemed to be sodium sensitive (SS), and if the summation <10 mm Hg, the subject was judged as non-sodium sensitive (NSS).

### Blood pressure measurement

Medical practitioners measuring blood pressure all received professional trainings based on WHO standards, and passed relevant examinations [12]. The environment for BP measuring was quiet and comfortable for subjects, who sat and rested quietly and did not drink anything. Cuffs of various sizes were chosen for subjects based on sizes of their upper arms, to measure their right brachial pressure in sitting position. The pulse disappearing pressure was taken first; it plus 30 mmHg would be the Peak Inflating Pressure. The formal measurement of BP started with increasing the pressure to Peak Inflating Pressure, and then the operator deflated at a speed of 2 mmHg per second. Following the Korotkoff sound method, the operator took the number when the first sound appeared as the SBP, and the number when the fifth sound appeared as the DBP. BP was measured three times for each subject, with interval of 30 seconds between each measurement; average SBP and DBP of the three measurements were calculated. MABP = 1/3SBP + 2/3 DBP.

### Statistical method

Statistical analysis was performed using the Statistical Package for the Social Sciences (SPSS 13.0). One-way ANOVA was used as appropriate to evaluate differences in the mean values of specified variables between groups, differences in proportions between two groups were tested by χ2 analysis. Age, sex and body-mass index (BMI) were adjusted in multivariable analysis. Two-tailed values of P < 0.05 were taken as statistical significant. All data were expressed in terms of mean ± s.d.

## Results

### Salt sensitive adolescents cohort follow Up

There were 310 baseline samples (101 SS subjects and 209 NSS subjects). 223 subjects were followed (77 SS and 152 NSS) and 84 were lost (54 working in other places, 7 studying in other places, 11 relocating for marriage, 4 moving to other places, 2 serving the army, 6 not found). Therefore, the response rate at follow up for surviving baseline samples was 71.9%, with 70.3% for SS subjects and 72.7% for NSS subjects. Table 2 showed that there was no significant statistical difference between SS group and NSS group in terms of age, body mass index, blood glucose or blood lipid (P > 0.05).

**Table 2:** Descriptive statistics for cohort subjects in SS and NSS at follow-up in 2005 (x±s)

**Group**	**Sample No. (Male/Female)**	**Age (Year)**	**BMI (kg/m**^**2**^**)**	**Fasting Blood Glucose (mmol/L)**	**TC (mmol/L)**	**TG (mmol/L)**	**HDL-C (mmol/L)**	**LDL-C (mmol/L)**
SS	71 (38/33)	27.4±1.8	22.5±3.0	4.6±0.6	4.4±0.8	1.4±0.6	1.0±0.2	2.6±0.6
NSS	152 (82/70)	27.2±1.8	21.9±2.8	4.7±0.5	4.4±0.7	1.3±0.6	1.1±0.2	2.6±0.4

SS, Salt Sensitive; NSS, Non-salt-sensitive; BMI, body mass index; TC, Total Cholesterol; TG, triacylglycerol; HDL-C, high density lipoprotein cholesterol; LDL-C, low density lipoprotein cholesterol.

### Analysis of blood pressure evolution in adolescents with SS and NSS

**Figure 1:**
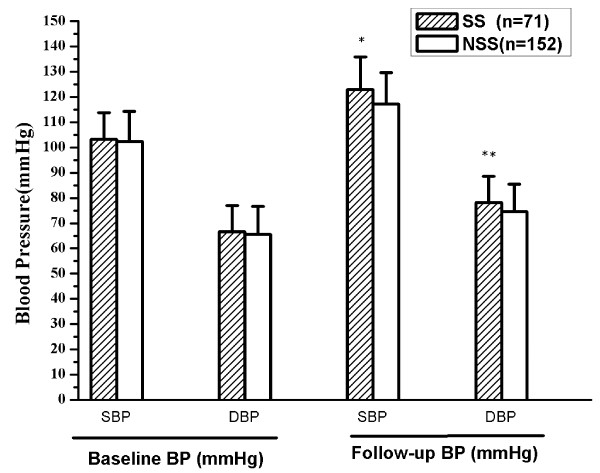
**Showed comparison of BP evolution by longitudinal analysis between SS and NSS adolescents.** After 18 years, BP of the SS group increased largely, with both SBP and DBP significantly higher than those in NSS group (SBP : *P*<0.01, DBP : *P*<0.05). Blood Pressure Evolution Followed up 18-Year in SS and NSS Groups. SS : Salt Sensitive NSS: Non-salt-sensitive SBP : systolic blood pressure DBP : diastolic blood pressure. Compared with SS group, * *P*<0.01 * *P<0.05.

Comparison of BP evolution between SS and NSS adolescents is shown in Figure 1. There was no statistical difference in baseline BP levels in the two groups. However, when followed up after 18 years, BP of the SS group increased largely, with both SBP and DBP significantly higher than those in NSS group (SBP : P < 0.01, DBP : P < 0.05).

### Incidence of hypertension in salt sensitive youth

At baseline, there was no statistical difference in SBP and DBP between the two groups. After 18 years, the follow-up study showed that, the incidence of hypertension (≥140/90 mmHg) in SS group was much higher than that in NSS group (P < 0.05) (Table 3).

**Table 3:** The incidence of hypertension in Salt Sensitive adolescents after 18 year in 2005

**Group**	**Sample No (Male/Female)**	**Baseline Age (Year)**	**Number of hypertension**	**Incidence of hypertension**	**RR (95% CI)**
SS	71 (55/44)	9.3±1.9	11	15.5%	2.34 (1.042-5.251)*
NSS	152 (118/91)	9.4±2.1	10	6.3%	

SS: Salt Sensitive NSS: Non-salt-sensitive Compared with SS group, *P<0.05.

## Discussions

The present study evaluated the evolution of blood pressure over 18 years with different BP salt-sensitivity from adolescents to youth. There was no significant difference in baseline BP of SS and NSS adolescents, the SS youth showed higher BP increases and significantly higher incidence rate of hypertension than NSS youth. Our findings indicate that adolescents with higher BP salt-sensitivity have a higher rate of incident hypertension in youth.

In 1970s, Luft and Kawasaki proposed the concept of SS based on hypertension patients BP changes and sodium retention responding to high salt intake. A person with salt sensitivity, if long-term exposing to high dietary salt environment, would lead to high blood pressure [5,13]. Literatures reported that SS tested varies in different nations and races, ranging from 5% to 25%, the founding in hypertensive patients and population with hypertension family history is about 50%-60% and which is comparatively higher in the senior and the black [5,13-15]. In our previous study, it was indicated that about 28% of adults in general population and 60% of hypertensives in China were determined as salt sensitivity [16]. Follow-up studies have reported that a salt-sensitive state is persistent and reproducible over time [17]. Furthermore, salt sensitivity was associated with higher incidence of cardiovascular events and increased mortality independent of blood pressure [18,19]. In the “New Definition of Hypertension (2005)” of American Society of Hypertension, SS was listed as the sign for early target organ damage by hypertension. Therefore, SS is an important factor in prevention of hypertension and cardiovascular disease.

The essential hypertension mostly originates from children. Following a cohort's BP evolution from their early years to youth has strategic meaning in identifying risk factors for essential hypertension, exploring its pathogenesis mechanism and exerting effective early prevention of hypertension [3]. There has been major progress in studies, examinations, prevention and treatment of adolescents’ and youth’s hypertension in recent years. The US has established a large-scale national database for regular BP of adolescents, which effectively helps identify and appraise adolescents with abnormal BPs. A study in Spain adolescents showed there was a significant association between salt perception and BP [20].However, current studies have not given the evidence on the relationship between sodium and blood pressure in early life [21]. The Hanzhong Adolescents Hypertension Cohort established in 1980s has been followed up for over 20 years. The current ages of the subjects in the cohort range from 25 to 35 years old, which means that their BP tend to be “steady”. Therefore, this is an important time point to explore the BP evolution from adolescents to adults and the relevant risk factors for hypertension.

A study in a group of American black youth with normal BP showed that, SS subjects with hypertension family history showed significantly higher BP responses to both sodium load and psychological stimulus, indicating that SS might be the genetic risk factor for adolescents’ hypertension pathogenesis [22,23]. Recently, a 15-year follow-up study conducted by Barba on a cohort of subjects averagely aged 46 with normal baseline BP showed that, there was significantly higher hypertension incidence rate in SS subjects than in NSS subjects. The logistic regressive analysis indicated that SS is an independent risk factor for pathogenesis of hypertension [8]. To our knowledge, this is first long-term prospective cohort follow-up study to investigate the predisposing of SS adolescents to essential hypertension in future. These indicated that SS is independent risk factor for adolescents' hypertension pathogenesis and SS adolescents’ high salt intake from childhood plays a determining role in evolution of their BP in later years. Low salt diet, even if as early as in children, may be the the best approach to the prevention of hypertension.

### Limitations

The sample under study was composed of Chinese people only, thus, our findings cannot be extrapolated to other ethnic groups.

The response rate at follow up for surviving baseline samples was 71.9%, with 70.3% for SS subjects and 72.7% for NSS subjects. The response rate was slightly low.

In conclusion, the present study provides long-term prospective data on the incidence of hypertension with different BP salt-sensitivity from adolescents to youth. These indicated that SS is independent risk factor for adolescents' hypertension and SS adolescents’ high salt intake from childhood plays a determining role in evolution of their BP in later years. These results provide further support to the role of salt-sensitivity and dietary salt intake in the pathogenesis of hypertension.

## Abbreviations

SS: Salt sensitive; NSS: Non-salt-sensitive; BP: Blood pressure; BMI: Body mass index; MABP: Mean arterial blood pressure; SBP: Systolic blood pressure; DPB: Diastolic blood pressure.

## Competing interests

The authors declared no conflict of interest.

This study was supported with grants from the National Program on Key Basic Research Project of China (973 Program, 2012CB517804) and Natural Science Foundation of China ( NO: 81070218 and 30671160).

## Authors’ contributions

Jianjun Mu and Zhiquan Liu conceived of the study, and participated in its design and coordination, especially for the baseline survery. Shuhui Zheng, Qiufang Lian and Fuqiang Liu participated the follow-up work, included BP measurements, blood and other information collected. Shuhui Zheng drafted the manuscript. Fuqiang Liu helped with data statistics. All authors read and approved the final manuscript.
